# Surgical Management of Pericardial Cysts: A Single-Center Retrospective Study

**DOI:** 10.7759/cureus.49298

**Published:** 2023-11-23

**Authors:** Wei-min Zhang, Abdunabi Maimaitiaili, Rehemutulajiang Aizezi, Kadeyanmu Abulimiti, Fei Yan, Qiang Huo

**Affiliations:** 1 Department of Cardiac Surgery, The First Affiliated Hospital of Xinjiang Medical University, Urumqi, CHN

**Keywords:** surgery, pathology, imaging, mediastinal cyst, pericardial cyst

## Abstract

Introduction

Pericardial cysts (PCs) are infrequent, non-malignant, and congenital abnormalities. The identification and treatment of PCs remain a significant challenge, with limited research on surgical management.

Methods

We performed a retrospective study of patients with PCs who underwent surgical intervention at the First Affiliated Hospital of Xinjiang Medical University from February 2002 to December 2022.

Results

A total of 55 patients underwent surgery due to PCs during the study period. Thirty-one were females and 24 were males. The average age of the patients was 44.7 ± 12.9 (six to 63 years old). PCs were located in the right hemithorax in 50 (90.9%) patients and left hemithorax in five (9.1%) patients. Approach methods were video-assisted thoracoscopic surgery (VATS) in 43 (78.2%) cases; thoracotomy in 11 (20%) cases, and median sternotomy in one (1.8%) cases. The average postoperative hospitalization period was 5.6 days (two to 14 days). Three patients developed postoperative complications (two pleural effusion, one pneumonia), whereas no mortality was observed in any patient due to the operation. Forty-one patients (74.5%) were followed up for three months to eight years postoperatively, during which no recurrent cysts were detected.

Conclusion

In this single-center retrospective study, we demonstrated that pericardial cyst cure is an appropriate operation according to cyst characteristics. VATS has been shown to be highly effective and safe in patients with PCs, offering the advantage of reducing postoperative morbidity efficiently.

## Introduction

Pericardial cysts (PCs) are rare and benign lesions located in the mediastinum. The incidence rate of PCs is one in every 100,000 persons, and they account for approximately 6% of mediastinal masses [1]. Pericardial cysts can be found anywhere in the pericardium. The frequency of involvement of the right cardiophrenic angle (51%-70%) is high, followed by the left cardiophrenic angle (22%-38%) or the superior part of the mediastinum (8%-11%) [2]. PCs are usually asymptomatic and found incidentally on imaging examinations [3,4]. Clinical symptoms occasionally result from compression of adjacent structures or other complications. The identification and treatment of PCs present difficulties due to the wide range of presentations and limited clinical data available.

Conservative and surgical modalities are used in PC treatment [5]. The main indication for surgical intervention includes symptomatic PCs, and the management of asymptomatic PCs is still debated. However, literature concerning surgical experiences with PCs is limited. This study aims to identify the characteristics and outcomes of patients who have undergone surgical resection of PCs at our hospital over the past two decades.

## Materials and methods

Study design

Cases of 55 patients undergoing surgical treatment for pericardial cysts between February 2002 and December 2022 were retrospectively reviewed. Diagnostic workup was performed based on the different imaging modalities, such as echocardiography, chest radiography, computed tomography (CT), and magnetic resonance imaging (MRI). All PCs were diagnosed by pathological confirmation after surgical resection. Patients with mediastinal cysts who were not managed surgically were excluded from the study. Moreover, patients were examined in terms of symptoms, diagnosis method, cyst location, surgical interventions, and outcomes.

Surgical procedure

All patients were administered general anesthesia with endotracheal intubation. The patients underwent video-assisted thoracoscopic surgery (VATS) with a double-lumen endotracheal tube to allow ipsilateral lung collapse and single-lung ventilation. The selection of the surgical strategy was based on factors, such as the number, size, location, and complexity of the cyst. Prior to 2013, the majority of surgeries were conducted through thoracotomy, whereas VATS became the routine approach after 2013. The surgical approaches employed included thoracotomy in 11 cases, VATS in 43 cases, and median sternotomy in one case. After entering the thoracic cavity, a thorough thoracic exploration of the cyst position, freedom, and relationship with the surrounding tissues, was initially performed. The boundary was determined between the cyst and the surrounding tissues. The cyst was separated from the surrounding tissues with a combination of blunt and sharp methods. The operation was cautiously performed to avoid major blood vessels, neural structures, and vital organs. Most cysts were removed intact. In some cases, a large cyst was difficult to mobilize and remove completely without causing damage to the surrounding tissue. The use of cyst aspiration during surgery may reduce the tension of the cyst and is helpful to separate the cyst from the surrounding tissue.

## Results

This study included 55 patients suffering from PCs, 24 males and 31 females, with an average age of 44.7 ± 12.9 years (ranging from six to 63 years old). A total of 33 patients (60%) had symptoms, the most common of which were chest pain (14, 25.5%), chest distress (12, 21.8%), cough (six, 10.9%), and palpitations (one, 1.8%). Twenty-two patients (40%) were asymptomatic, and their PCs were discovered by accident during an imaging examination.

All patients were diagnosed with echocardiography, chest radiography, and CT. Only one patient underwent MRI. PCs were detected in only 13 of the cases by echocardiography, with a 23.6% positive rate. All cyst lesions were clearly visible by CT or MRI in 100% of cases.

Fifty (90.1%) patients had pericardial cysts in the right hemithorax, and five (9.1%) had cysts in the left hemithorax. Of the 55 cysts, 35 were located in the right cardiophrenic angle, 10 in the right upper-middle mediastinum, one in the right posterior mediastinum, four in the front of trachea and closely attached to the superior vena cava and the ascending aorta, two in the left cardiophrenic angle, one in the left ventricular apical, one in the left upper-middle mediastinum, and one in the left posterior mediastinum. Cyst sizes varied, ranging from 2 cm to 12 cm in diameter. Furthermore, five cysts showed compression on the adjacent cardiac structures, which was confirmed by surgery. The involved structures included right ventricle (one), pulmonary artery (one), ascending aorta (one), and bronchus (two). Abdominal ultrasound or CT revealed seven cases with abdominal cysts: three cases had simple renal cysts, two cases had multiple cysts in the liver and kidney, one case had multiple liver cysts, and one case had adrenal cyst.

All the patients underwent surgery at our department of cardiac surgery and thoracic surgery. Approach methods were thoracotomy in 11 (20%) cases, VATS in 43 (78.2%) cases (41 right and two left), and median sternotomy in one (1.8%) case. The average postoperative hospitalization period was 5.6 days (two to 14 days). Postoperative complications occurred in three patients (two pleural effusions, one pneumonia), but no mortality was observed in any case. Forty-one patients (74.5%) were followed up for three months to eight years postoperatively, and only 12 patients (21.8%) were followed up for longer than 24 months, during which no recurrent cysts were detected.

**Figure 1:**
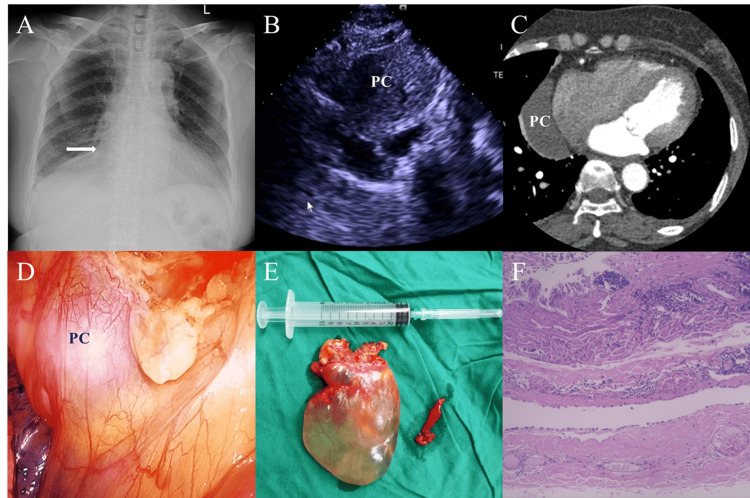
Typical case of pericardial cyst (PC). A. Chest radiograph shows soft tissue density along the right heart border (white arrow). B. Transthoracic echocardiography shows a huge cystic structure in the right cardiophrenic angle. C. Computed tomography shows a thin-walled, non-enhancing cystic mass arising from the pericardium. D. Thoracoscopy shows a hemispherical, pale and smooth-faced cyst. E. Gross specimen of the pericardial cyst after excision. F. Histological examination revealed the lumen of a cyst lined by a layer of mesothelial cells.

Herein, we describe a typical case of a 57-year-old woman who complained of palpitation for six months. During the evaluation, an incidental finding of a pericardial cyst measuring approximately 9 cm x 3.5 cm was discovered. The patient underwent VATS for excision of the PC. She recovered well and was discharged three days after surgery. No evidence of recurrence of the disease was found after 15 months. All relevant radiological, operative, and pathology data of the case are presented in Figure 1A-1F.

## Discussion

Pericardial cysts are uncommon mediastinal abnormalities. PCs are caused by an incomplete coalescence of fetal lacunae during the development of the pericardium [6]. Despite that PCs can occur anywhere in the mediastinum, the right cardiophrenic angle is the most common site. Histologically, the cyst wall is typically composed of a single layer of mesothelial cells and filled with clear fluid. The lesions are usually well-defined, round or elliptical in shape, with thin walls and no internal septation. In addition, PCs are acquired as a complication of inflammatory conditions, pericarditis, and surgery [7]. Generally, they are unilocular and smooth, with varied sizes (2-28 cm) [8].

PCs are usually asymptomatic and found incidentally during a routine radiological examination. Depending on the cyst size and anatomic location, the clinical presentation relies heavily on the pressure generated by the cyst on adjacent structures. PCs can present with chest pain, dyspnea, cough, syncope, and arrhythmia [9-11]. They are usually detected between the age of 30 to 50 years with no gender preference [12]. Among our patients, the mean age was 44.7 ± 12.9 years, and 56.4% involved women.

A majority of PCs are detected through imaging examination, including echocardiography, chest radiography, CT, and MRI [13]. Echocardiography is useful not only for identifying cysts but also in assessing complications associated with them. Compared with other imaging modalities, it has an incomparable advantage in detecting intracardiac lesions together with the cysts. In addition, it can distinguish between other possible diagnoses, such as a prominent fat pad, a prominent left ventricular aneurysm, an enlarged left atrial appendage, an aortic aneurysm, or a solid tumor [14]. However, echocardiography has low sensitivity in detecting PCs because of the obstruction of the skeleton and lung tissue in the acoustic window [3]. Transesophageal echocardiography can prove to be useful when transthoracic echocardiography is insufficient to determine a diagnosis [2]. CT is widely accepted as an essential examination; it shows the precise location and relationship between adjacent structures as well as an excellent delineation of pericardial anatomy. Some cysts contain non-serous fluid that MR can detect better than CT by determining their fluid nature.

Differential diagnoses of mediastinal masses include bronchogenic cysts, lymphangiomas, aortic aneurysms, ventricular aneurysms, neurogenic tumors, teratoma, and pericardial fat pads [15]. However, CT and MRI cannot differentiate between pericardial and epicardial cysts [16]. Moreover, only a histopathological examination of the tumour is conclusive [17]. In this study, three patients were misdiagnosed with bronchogenic cysts based on imaging studies prior to operation.

The majority of PC cases follow a benign clinical course, although some complications have been reported, including pericarditis, cyst rupture, cardiac tamponade due to rupture or hemorrhage, right heart obstruction, erosion of the cyst into the superior vena cava and right ventricular wall, heart failure, arrhythmia, bronchus obstruction, syncope, and even sudden death [18].

Management options for PCs are abundant, depending on their symptoms and characteristics. Small or asymptomatic PCs can be managed conservatively, but long clinical and imaging regular follow-ups are required [19]. During the follow-up, spontaneous resolution of pericardial cyst has also been observed [20]. Although several mechanisms regarding the spontaneous resolution of these cysts have been proposed, the best-supported hypothesis at this time is cystic rupture into the pleura [2].

Surgery is generally recommended when cysts are enlarged and patients have symptoms associated with PCs or their diagnosis is uncertain [21]. Surgery strategy is proposed due to the potential risk of malignant transformation, cyst infection, cyst rupture, compression, or erosion of adjacent structures. Several treatment options include percutaneous aspiration, ethanol sclerosis, or resection via VATS, thoracotomy, or median sternotomy [5]. The procedure should be performed according to the characteristics of the pericardial cyst to achieve a curative effect. Percutaneous treatment of PCs with needle aspiration has been increasingly reported in recent years [22,23]. However, it cannot perform a histopathological diagnosis. Possible complications include hemorrhage, infection, and recurrence [7]. If this approach is not feasible, then thoracoscopic resection is the best and preferred treatment option for PCs [24], because it is less traumatic, less painful, faster to recover from, and less expensive than traditional surgery. As thoracoscopy experience increased, thoracotomies became less common. With today’s video-assisted surgery, open thoracic surgery is only indicated in a few cases with complicated anatomy or extensive adhesions, mostly caused by inflammation or infection. In the last few years, VATS has become a procedure of choice in our institution. Our group of 43 patients (78.2%) underwent VATS, without serious postoperative complications recorded.

Complete removal of the cyst wall is recommended for pathological analysis and to prevent recurrences [19]. Incomplete resections can lead to recurrence because some cysts have adhered tenaciously to the trachea, bronchus, esophagus, or other organs. In some cases, large cysts were difficult to mobilize and remove completely without damaging the adjacent structures. The use of cyst aspiration during surgery may reduce the risk of rupture of the cyst [2]. Prior to cyst aspiration, efforts should be exerted to avoid the potential spread of infection or malignancy in the thoracic cavity during cyst aspiration.

Limitations

The primary constraint of our study is the limited sample size. We encountered a low follow-up rate within a small cohort of patients, with only 74.5% (41) out of 55 patients being followed up. Furthermore, only 12 patients (21.8%) were monitored for a duration exceeding 24 months. Consequently, the two-year follow-up period may be deemed inadequate for evaluating long-term postoperative prognoses or recurrence. Extensive multi-centric trials should be conducted to enhance the association.

## Conclusions

PCs are extremely rare and benign in nature, with the primary concern for surgery being patients exhibiting symptoms and those at potential risk of clinical deterioration. Our study has demonstrated that surgical intervention for pericardial cysts is an appropriate approach, depending on the characteristics of the cyst. VATS can be safely and effectively utilized in the management of pericardial cysts. Furthermore, thoracoscopic cyst resection provides the opportunity for histological examination in relevant cases.
